# A successful bilateral iliac arteries ligation as an emergency management to a life-threatening AVM bleeding: A case report and literature review

**DOI:** 10.1016/j.ijscr.2023.108022

**Published:** 2023-03-28

**Authors:** Dema Adwan, Wessam Taifour, Fatima Haj Reslan

**Affiliations:** Obstetrics and Gynecology Hospital, Damascus University, Damascus, Syria

**Keywords:** Uterine arteriovenous malformation, Bilateral internal iliac artery ligation, Compression sutures, Trans Vaginal Sonography with Doppler, Case report, Literature review

## Abstract

**Introduction and importance:**

Uterine arteriovenous malformation (AVM) is a defect due to direct connection between uterine arteries and veins, it has wide range of symptoms and severity, usually causes vaginal bleeding which may be mild or severe and may cause death in some rare cases. Diagnostic methods may include ultrasound, MRI, CT and/or angiography which reveal a high blood flow hypoechoic mass. Many options have been applied as management procedures, including invasive and noninvasive procedures, aiming to save patient life and stop bleeding or preserve fertility in the less severe cases.

**Case presentation:**

21 years old primigravida patient developed episodes of severe vaginal bleeding due to AVM after complete molar pregnancy evacuation and chemotherapy, managed successfully by bilateral internal iliac artery ligation and applying compression sutures on the uterus and continuing with compound medical treatment, trying to preserve fertility and ability to get pregnant in the future.

**Clinical discussion:**

We point out AVM types, its sings, symptoms and the diagnostic procedures. We discuss the bilateral iliac artery ligation and applying modified compression sutures on uterus as possible emergency managements to AVM when there is a threatening on life due to huge bleeding. We also mentioned the other surgical and medical treatments.

**Conclusion:**

We confirm the importance of keeping AVM in mind as a possible diagnose when there is unknown cause vaginal bleeding. Consider doing bilateral iliac artery ligation and applying the modified compression sutures as emergency procedures to stop or reduce AVM bleeding.

## Highlights

• Uterine arteriovenous malformation is a rare condition where there are abnormal connections between arteries and veins.
• Transvaginal color Doppler ultrasound is a suitable way to put diagnose.
• AVM treatment's options depend on patient's hemodynamic status and on the desire of preserving fertility.
• Bilateral iliac artery ligation is one of AVM treatment's options especially in emergency situations.

## 1 Introduction

AVM (arteriovenous malformation) is a case where there are abnormal connections between arteries and veins with the absence of capillary vessels, leading to direct blood flow from arterial system to the venous system with a high flow and high pressure gradient [1]. It may cause large vaginal bleeding and even life-threatening bleeding in the severe conditions or may be asymptomatic. There are two types of AVM, acquired which is more common and may be a result of cesarean section, gestational trophoblastic neoplasia, after curettage or cervical, endometrial neoplasia and congenital form result from embryonic differentiation failure [2], [3]. Many AVM cases may pass undiagnosed or misdiagnosed, so it is difficult to determine a true and accurate incidence rate [3]. Echo Doppler, CT, MRI or angiography may be used in making diagnose.

Treatment depends on the desire of preserving fertility, hysterectomy is the selective option when there is not an intention of being pregnant, otherwise we must choose a conservative treatment as medication, artery embolization and internal iliac artery ligation [3].

Our work has been reported in line with the SCARE criteria [4].

## 2 Presentation of case

**Fig. 1:**
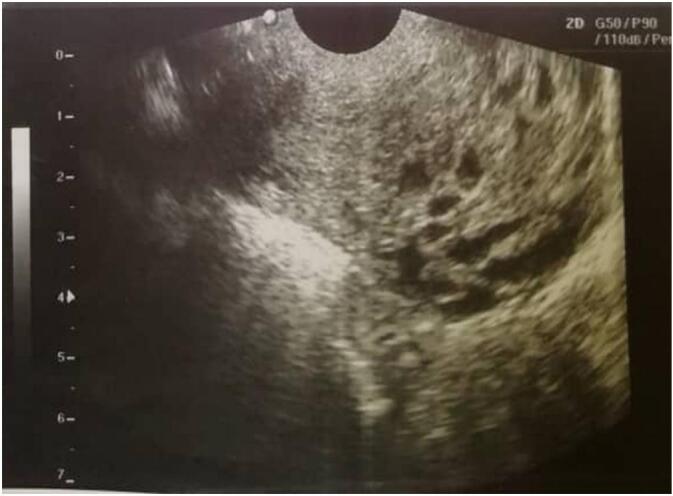
Large uterus consist with molar pregnancy consist with invasive molar pregnancy.

A 21 years old primigravida woman was referred to our hospital after a molar pregnancy evacuation at 9 weeks gestation in another hospital two months before. Her main complaints were continuous vaginal bleeding after the evacuation for two months unresponded to medications and general fatigue. Her blood pressure was 100/70 mm Hg and the heart rate was 98 beats/min, laboratory tests: Hb: 9.6 g/dl and B-HCG level = 72,194.12 mIU/mL. The patient had no significant medical and family history, she denied alcohol and tobacco use. The ultrasound showed large uterus (Fig. 1), a whole body CT scan was done and revealed a 5.5 × 6.5 cm heterogenous mass with unclear borders close to the posterior wall of an exaggerated uterus.

The patient was hospitalized with the diagnosis of invasive molar pregnancy and received three high risk courses of chemotherapy, the B-HCG levels reduced then from 72,194.12 mIU/mL to a negative value in 5 months.

**Fig. 2:**
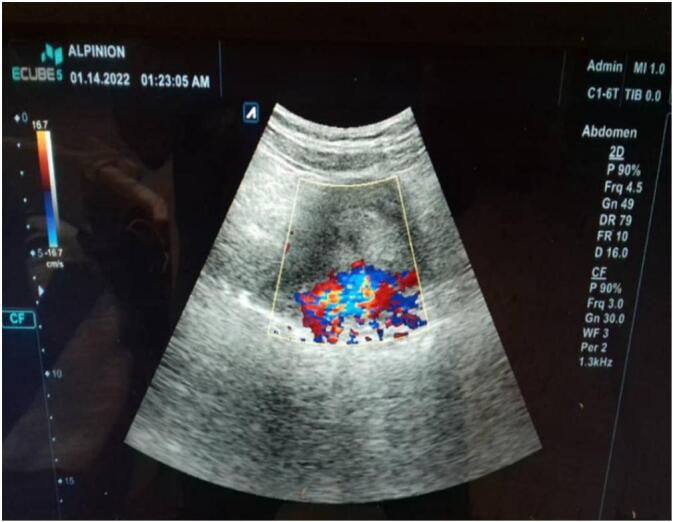
Trans Vaginal Sonography with Doppler showing an AVM mass behind the uterus.

**Figs. 3–4:**
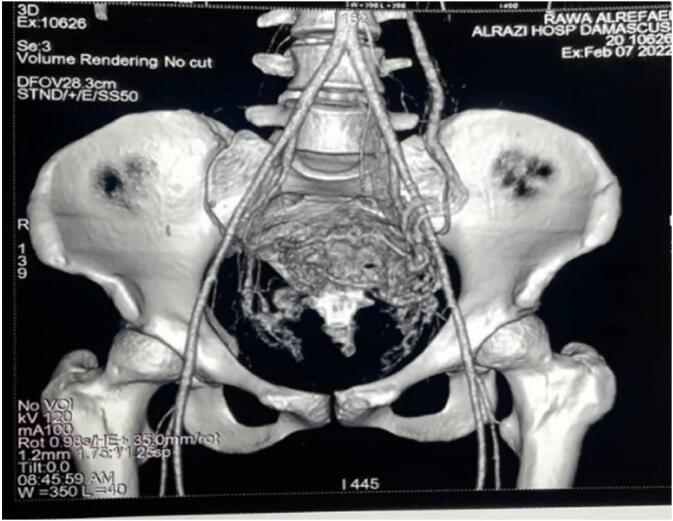

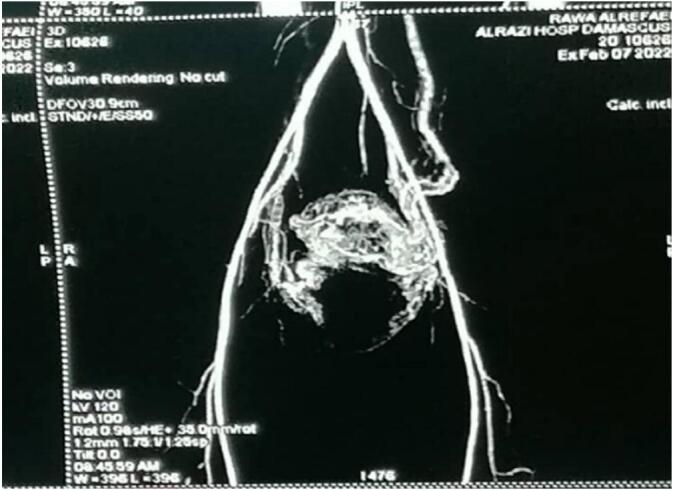
Bilateral arterial and venous uterine fistulas from the bilateral internal iliac arteries by computed tomography.

The patient admitted again in the hospital 5 months later with general fatigue and moderate vaginal bleeding, the HGB = 7.3–PT = 62 %. Five units' whole blood and five plasma units have been transmitted within two days. A Trans Vaginal Sonography with Doppler was made and revealed a heterogeneous mass mostly represent an AVM mass behind the uterus (Fig. 2). A computed tomography angiography revealed sings of bilateral arterial and venous uterine fistulas from the bilateral internal iliac arteries (Figs. 3–4).

The patient was discharged home stable with HGB = 9.9 and was put on continuously combined oral contraceptive pills.

She was admitted in the hospital again 25 days later with severe vaginal bleeding and HGB = 5.4, her pulse was 130 and the blood pressure was 100/60 mm Hg, she was tubed in the intensive care unit and whole 6 blood units plus six plasma units were transmitted. Another episode of severe vaginal bleeding occurred to almost of 1.5 L of blood after few minutes later; therefore the decision of surgery was made.

**Fig. 5:**
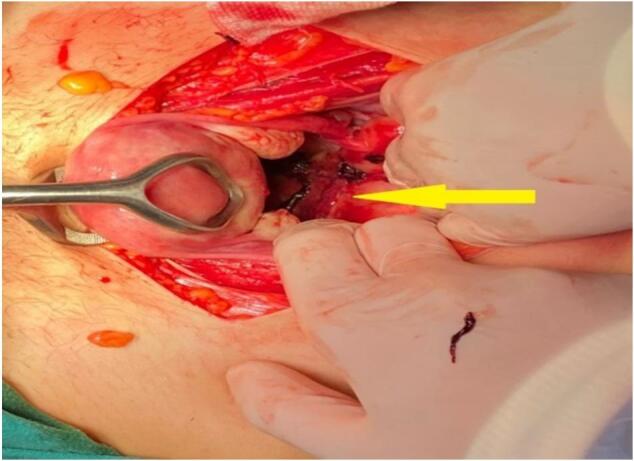
A vascular mass behind the uterus (yellow arrow) during surgery.

Open laparotomy under general anesthesia was done by our staff where we found a vascular mass behind the uterus, in front of rectum behind the peritoneum measuring 5 × 6 cm (Fig. 5), we made a bilateral internal iliac ligation and compression modified sutures on the uterus (we ligate the thread of Vicryl 1 USP after taking off its needle to the thread of nylon with straight needle (by this way the nylon straight needle can penetrate the whole thickness of the uterus) and we made two vertical and two transverse sutures by the Vicryl thread only to form a square like shape that squeezed the uterus and reduced its size).

She was discharged home after one week of observation, 5 whole blood units and 15 plasma units were transfused, we gave her single dose of depo provera IM on day 2 after surgery and yasmin tablets for 5 days.

**Fig. 6:**
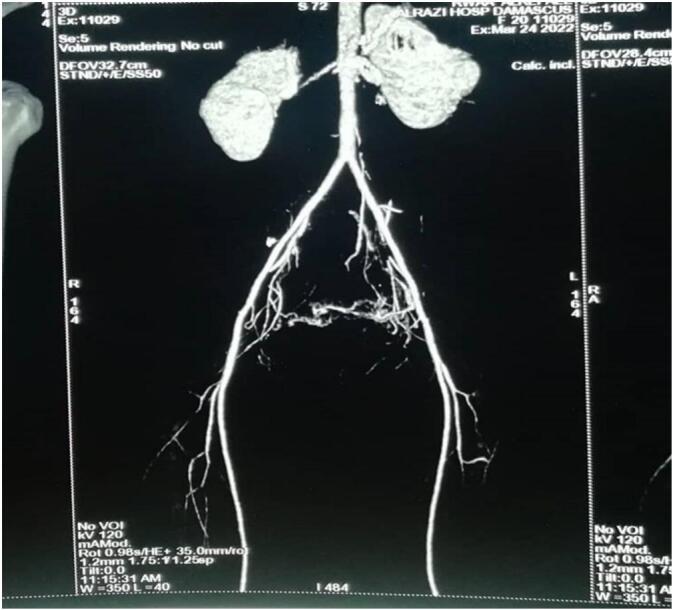
Computed tomography angiography shows absence of the past fistulas.

**Fig. 7:**
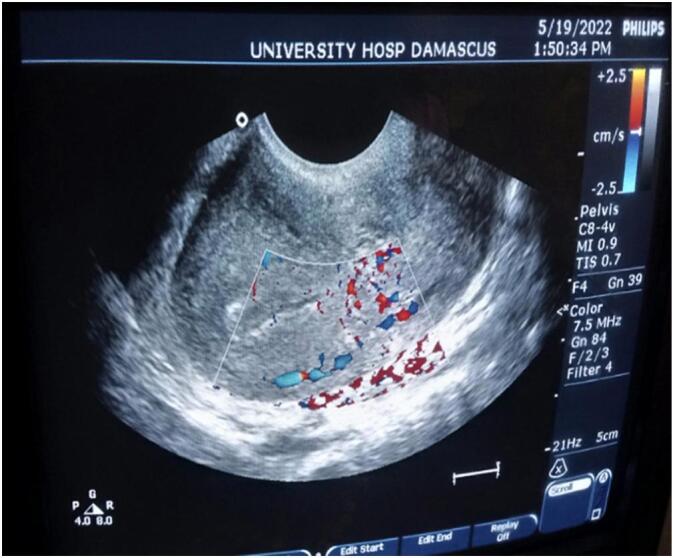
No obvious vascular malformation behind the uterus on TVS with Doppler.

Serial later TVS with Doppler showed no obvious vascular malformation behind the uterus, a CT 40 days after surgery did not show the previous vascular malformation (Fig. 6, Fig. 7).

## 3 Discussion

AVM is a rare condition usually occurs at fertile age, with no accurate incidence rate because some cases may be misdiagnosed or went undiagnosed and resolved spontaneously [1].

There are two types of AVM: congenital AVM which results from abnormal embryologic development of primitive vascular structures, which lead to abnormal connections between arteries and veins and may invade surrounding structures and can involve anybody's tissue [5], and traumatic or acquired AVM that results after interventions and trauma on uterine such as: dilation and curettage (D&C), therapeutic abortion, uterine surgery, infection, cesarean scar pregnancy, endometrial and cervical carcinoma and gestational trophoblastic disease [6], [7].

The AVM in this case was mostly developed after a complete molarian pregnancy which was treated by evacuation and chemotherapy, even so it may be congenital AVM which activated and gave sings after pregnancy which increases the vasculature of uterus and after chemotherapy which may make vessels walls weaker.

AVMS may be existed or developed without presenting any symptom and could regress spontaneously or may cause abnormal vaginal bleeding ranges from mild to severe and potentially life-threatening or may lead to high cardiac output state, cardiac dysfunction and congestive heart failure [1], [2], [3], [4], [5], [6].

In our case the patient had experienced life-threatening and heavy episodes of bleeding that needed whole blood transfusion and intensive care unit admission and emergency treatment finally.

Different means can be used to diagnose AVM; even so it is challenging to be diagnosed accurately, 2-dimensional grayscale ultrasound scanning could reveal irregular tortuous tubular hypoechoic lesions, showing increased vascularity and turbulent high velocity blood flow when using color Doppler and most recently using transvaginal ultrasound scanning, which can now be used successfully in diagnosing and following up after treatment and in determine the severity of the case depending on PSV (peak systolic velocity) values in the range of 40–100 cm/s and the resistance index of the vessel ranging from 0.25 to 0.55, although uterine angiography was the gold standard diagnostic method in the past, but it is rarely used today to make diagnose unless using it in treatment [8], [9], CT and MRI can be also used.

We used color Doppler which revealed a high vascularity mass behind the posterior wall of the uterus; we did a computed tomography angiography in order to obtain further information about the lesion and its feeding vessels.

Despite the importance of the gynecological AVM, till now there is no clear algorithm or specific guidelines to handle with it, all the procedures were based on individual cases-mainly according to the clinical state [8].

So, when the patient is hemodynamically stable, the medical treatment can be tried even though there are no strict recommendations to give medicine or to determine the dose. We can categorize it into hormonal, uterotonics, methotrexate. A systemic review has conducted on 121 premenopausal women and found that the success rate was 88 % in general (progestins 82.8 %, methotrexate 90.9 %, uterotonics 100 % and danazol 66 %). The medical treatment has many good points, it costs less, noninvasive and has no effect on fertility [9].

Another option which has a wide application is uterine artery embolization. A systemic review by Daniel J and his collages included 54 patients found that the TCE (transcatheter embolization) primary success was 61 % and reached 91 % when the procedure needs to be repeated. There were no lines to follow after the recurrence of bleeding; some patient gets benefits of medicines, surgery or re-embolization which was discussed to embolize the contralateral side or the same side. Nevertheless, the clinical state plays main role in this decision [10].

Unfortunately, we didn't have this option because of the limitation of sources in our country, the high cost and the rapid deterioration in patients' clinical state.

To handle with a uterine AVM surgically we should first understand it with a vascular surgery look. The AVM consists of the nidus which connects the feeding arteries and the draining veins and if we do proximal occlusion with any intervention without penetration the nidus it is likely to develop new collateral feeders [11].

However, many procedures were done successfully without doing embolization first, but we noticed that they removed the AVM, not only doing ligation to the feeding artery. Ncol and his colleges ligated the feeding vessels above and below the uterine plexus which was along the left uterine wall in the broad ligament also between the plexus and the uterine wall without opening the myometrium, leading to successful pregnancy [12]. Similarly, in another case bilateral uterine artery ligation has been done and sutures were put directly into the AVM on the anterior wall of the uterus without interrupting the uterine cavity, not only the AVM resolved but the patient menstruated too after 8 months later [13].

In another surgical procedure, the uterine myometrial lesions were resected, and the uterus was reconstructed with an intact uterine cavity beside vessels' ligation, the vaginal bleeding stopped but unfortunately, she didn't menstruate later because of the uterine cavity adhesions [14].

During following up of patient who received chemotherapy due to invasive gestational trophoblastic tumor and who developed large arteriovenous malformation handled laparoscopically with bipolar coagulation of the ovarian and uterine arteries and surgical resection of the tumor. The defect in the uterine wall with an intact uterine cavity was reconstructed using sutures. The patient menstruated regularly after that [15].

Well, sometimes it is difficult to remove the AVM surgically, like in a case where the decision was to do ligation to the feeding arteries in attempting to resolve the AVM. The patient was scheduled to perform abdominal hysterectomy because of uterine AVM but because of the risk of massive hemorrhage from the expanding vessels the hysterectomy was canceled and only the main 6 arteries feeding the AVM, the uterine and ovarian arteries, and collaterals originating from internal iliac artery were ligated and resolution of the AVM has occurred [16].

Another similar case considered the internal iliac artery ligation by open laparotomy as a management of uterine AVM [17].

As the internal iliac artery is the main vascular supply of pelvic visceral structures so during peripartum hemorrhage or massive pelvic bleeding bilateral ligation of these arteries can reduces the pelvic arterial blood flow and pulse pressure.

However, most of the surgical interventions which included ligation of the internal iliac arteries or the uterine arteries were after an unsuccessful embolization of these arteries or when it was difficult to do the embolization.

Levy-Zaubermann et al. reported completely regression of the AVM after laparoscopic internal iliac artery occlusion with nonresorbable clips placed on both internal iliac arteries and both round ligaments after unsuccessful 2 bilateral embolization procedures to the both the right and left uterine arteries because angiography had showed vascularization of the uterine AVM via these two arteries [3].

The retrogression in the AVM after ligation of the feeding vessels only might be explained by the thrombosis which occurred in AVM mass after the ligation surgery and subsequent decreased in blood flow [18].

Actually, in our case we made the same somehow, after the decision was made to do open laparotomy because of the emergency situation, we did bilateral internal iliac ligation without interruption of the AVM directly and we did a modified compression sutures on the uterus as a method to decrees hemorrhage but these sutures did not include AVM mass because of the fear of rupturing the AVM and causing severe bleeding due to its fragile doughy texture.

After the surgery the bleeding has stopped and a massive blood transfusion was done to restabilize the patient. We did the surgical treatment not because of continuing vaginal bleeding which was stopped after surgery but as an attempt to prevent another episode of possible bleeding. However, we weren't sure about the succeeding of our procedure. So we gave her a depo provera injection and combined oral contraceptive pill for 5 days the day after surgery. A pelvic Doppler week later and a CT after 40 days revealed complete resolution of the previous AVM.

Currently, hysterectomy is indicated only for those women who do not want a child anymore and have difficulties in accessing the medical facilities [19].

Preserving fertility after AVM treatment especially after embolization is still questionable and there are no studies reports data on pregnancy rates after these treatments [20]. In our case the patient has menstruated normally after stopping medications, hopping that a pregnancy may occur in the future.

We still need further information and studies to determine the best management procedure to reduce and stop bleeding, resolve the AVM mass and preserve the uterus and fertility.

## 4 Conclusion

AVM can follow molar pregnancy and traumatic procedures on uterus, so it should be considered in every woman comes with abnormal uterine bleeding after molar pregnancy treatment procedures.

Transvaginal ultrasound scanning can be used successfully in diagnosing AVM and following it after treatment.

Bilateral iliac artery ligation should be considered in life-threatening situations when maintain fertility is preferable, and it could be shared with medical treatment.

A modified compression sutures could be applied on the uterus in order to reduce bleeding as a secondary and additional technique to the main procedure.

## Consent

Written informed consent was obtained from the patient for publication of this case report and accompanying images. A copy of the written consent is available for review by the Editor-in-Chief of this journal on request.

## Ethical approval

The study is exempt from ethnical approval in our institution.

## Funding

This research did not receive any specific grant from funding agencies in the public, commercial, or not-for-profit sectors.

## Guarantor

Wessam Taifour.

## Research registration number

Not applicable.

## CRediT authorship contribution statement

**Wessam Taifour:** study conception and design, data collection, writing the paper.
**Fatima Haj Reslan:** study concept, data collection, writing the paper.
**Dema Adwan:** study concept, Critical revision of the article.

All authors reviewed the results and approved the final version of the manuscript.

## Declaration of competing interest

We have no conflicts of interest to disclose.
